# Transboundary Cooperation in the Tumen River Basin Is the Key to Amur Leopard (*Panthera pardus*) Population Recovery in the Korean Peninsula

**DOI:** 10.3390/ani14010059

**Published:** 2023-12-22

**Authors:** Hailong Li, Puneet Pandey, Ying Li, Tianming Wang, Randeep Singh, Yuxi Peng, Hang Lee, Woo-Shin Lee, Weihong Zhu, Chang-Yong Choi

**Affiliations:** 1 Department of Agriculture, Forestry and Bioresources, College of Agriculture and Life Sciences, Seoul National University, 1 Gwanak-ro, Gwanak-gu, Seoul 08826, Republic of Korea; hai3456@snu.ac.kr (H.L.); krane@snu.ac.kr (W.-S.L.); 2 College of Geography and Ocean Science, Yanbian University, Yanji 133002, China; shadowlee@snu.ac.kr (Y.L.); 2184231366a@gmail.com (Y.P.); whzhu@ybu.edu.cn (W.Z.); 3 Research Institute for Veterinary Science and Conservation Genome Resource Bank for Korean Wildlife, College of Veterinary Medicine, Seoul National University, Seoul 08826, Republic of Korea; hanglee@snu.ac.kr; 4 Tiger and Leopard Conservation Fund in Korea, Seoul 08826, Republic of Korea; 5 National Forestry and Grassland Administration Key Laboratory for Conservation Ecology in the Northeast Tiger and Leopard National Park, Beijing 100875, China; wangtianming@bnu.edu.cn; 6 Ministry of Education Key Laboratory for Biodiversity Science and Engineering, College of Life Sciences, Beijing Normal University, Beijing 100875, China; 7 Amity Institute of Forestry and Wildlife, Amity University, Noida 201313, Uttar Pradesh, India; rsingh18@amity.edu

**Keywords:** Amur leopard, Tumen River basin, corridor movement, human disturbance, habitat degradation

## Abstract

**Simple Summary:**

The Lower Tumen River basin habitat at the Sino-North Korean border is crucial for reestablishing Amur leopards in the Korean Peninsula, where they once thrived. However, except for the Jingxin–Dapanling (JD) and Mijiang (MJ) corridors, most areas have become impassable due to human activities and urbanization. In this study, we evaluated the effectiveness of the MJ corridor by analyzing the species abundance, forest structure, landscape features, and disturbance factors. Our findings indicate that leopard activity is predominantly concentrated in the northern part of the corridor, with little to no presence in the middle and southern regions near the North Korean border. Human disturbances, forest structure, and infrastructural obstacles seem to impede the movement of leopards. To ensure the resurgence of the leopard population in the Korean Peninsula, it is imperative to mitigate or eliminate the impacts of these hindrances. This entails reducing human disturbances, enhancing forest structure, and removing infrastructural barriers. Such efforts are vital to facilitate the revival of the Amur leopards in their former range in the Korean Peninsula.

**Abstract:**

The interconnected forest regions along the lower Tumen River, at the Sino-North Korean border, provide critical habitats and corridors for the critically endangered Amur Leopard (*Panthera pardus orientalis*). In this region, there are two promising corridors for leopard movement between China and North Korea: the Jingxin–Dapanling (JD) and Mijiang (MJ) corridors. Past studies have confirmed the functionality of the JD corridor, but leopards’ utilization of the MJ corridor has not yet been established or confirmed. In this study, we assessed the functionality of the MJ corridor. The study area was monitored using camera traps between May 2019 and July 2021. We also analyzed 33 environmental and vegetation factors affecting leopard survival and analyzed leopard movement. In the Mijiang area, the Amur leopard was mainly active in the region adjacent to the Northeast China Tiger and Leopard National Park and did not venture into area near the North Korean border. The complex forest structure allowed leopards to move into the Mijiang area. However, the high intensity of human disturbance and manufactured physical barriers restricted further southward movement. Therefore, human-induced disturbances such as grazing, mining, farming, logging, and infrastructure development must be halted and reversed to make the Mijiang region a functional corridor for the Amur leopard to reach the North Korean forest. This necessitates inter-governmental and international cooperation and is essential for the long-term survival of the Amur leopard.

## 1. Introduction

The Amur leopard (*Panthera pardus orientalis*) is one of the world’s critically endangered large cats. It was once widespread across the Korean Peninsula, Northeastern China, and the Russian Far East [1,2]. Presently, the Amur leopard is isolated within the forest habitat of Russia’s southwestern Primorsky region (Land of the Leopard; 3690 km^2^) and the adjacent Jilin and Heilongjiang provinces of Northeast China (Northeast China Tiger and Leopard National Park (NCTLNP; 14,600 km^2^). According to a joint survey conducted by Chinese and Russian researchers in 2014–2015, the estimated population of Amur leopards in the two countries was 84 individuals [3]. Recent evidence suggests that the population of Amur leopards is recovering [4,5], but they still face severe conservation challenges such as inbreeding [6] and habitat loss [7]. Without intervention, under varying levels of inbreeding depression, the risk of extinction within 100 years ranges from 10.3% to 99.9% [8]. This risk can be mitigated by expanding their habitats and establishing larger populations within these territories [9].

**Figure 1:**
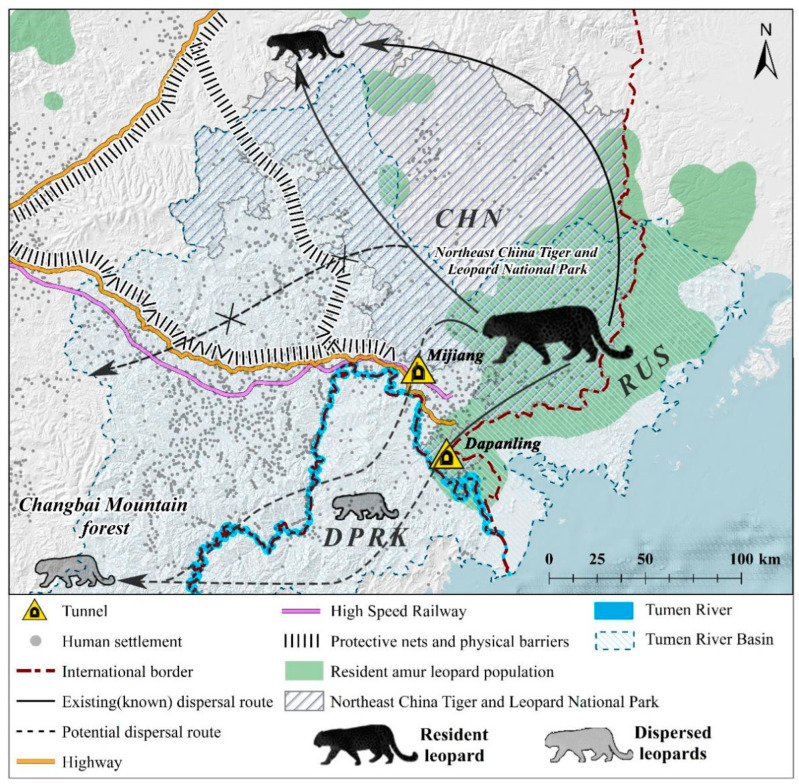
Distribution of the Amur leopard in Far East Asia (CHN—China, RUS—Russia, DPRK—North Korea). Note: Gray animals represent dispersed Amur leopards [7]. The solid black line and arrows depict the current dispersal path, while the dotted lines indicate possible dispersal paths for leopards [19,20].

The Tumen River basin, spanning 525 km and serving as a border river between China and North Korea, contains well-preserved forest ecosystems [10]. This region includes the Changbai Mountain Nature Reserve (CMNR) in the upstream area and NCTLNP in the downstream area (Figure 1). NCTLNP hosts the highest population density of Amur tigers and leopards in Northeast China. Monitoring results over the past two decades indicate that these animals are spreading westward within China’s territory, toward the Changbai Mountains (also known as Baekdu Mountains) [11]. While the Korean Peninsula and Changbai mountain region once supported a significant leopard population in their historical distribution, Amur leopards now rarely appear in the Sino-Korean border forests, except for a small part of the downstream area of the Tumen River [7,8,12]. Wildlife surveys conducted in the Changbai Mountain area between 1976–1977 and 1982–1983 reported 45 and 30 leopards, respectively. However, since the 1980s, leopards have disappeared in the Tonghua and Jilin areas [13]. According to the Red Data Book of DPRK (Animal), tigers and leopards have not been sighted in North Korea since 2000 [14].

In a questionnaire-based survey [15], residents of villages and hamlets in the Tumen River basin additionally informed us about recent tiger hunting on the North Korean side of the Sino-Korean border. This suggests that big cats can travel through the forests of the Sino-Korean border region to reach North Korean territory. There is a significant lack of research on how the Amur leopard moves between China and North Korea [16,17]. Apart from the Jingxin–Dapanling (JD) and Mijiang (MJ) corridors, the areas on the Sino-Korean border in the Tumen River basin have become physical barriers for leopards and other large mammals due to the presence of dense human settlements and protective nets on both sides of high-speed railways and highways (Figure 1). Leopards use the JD corridor for cross-border movement, as evidenced by camera-trap studies [18]. However, the functionality of the MJ corridor has not yet been confirmed.

Ensuring connectivity and the free movement of wildlife in the Tumen River basin is vital for preserving biodiversity, preventing species extinction, and facilitating the range expansion of endangered wildlife. Our goal is to assess the potential of the MJ corridor for leopard dispersal from China to North Korea and to identify issues affecting cross-border leopard movement. This research provides crucial references for establishing an effective cross-border animal dispersal corridor between China and North Korea.

## 2. Materials and Methods

### 2.1. Study Area

The study area is situated in the downstream region of the Tumen River, covering a total area of 256 km^2^ (N 42.90°–43.10°, E 130.02°–130.40°). Predominantly, the site is covered by a mix of coniferous (6.42%), broad-leaved (24.38%), and mixed (68.97%) forests, resulting in an impressive overall forest coverage of 92.94%. The terrain is characterized by medium to low mountains, generally below 1000 m above sea level (asl), with most of the landscape falling within the 200 m to 600 m asl range. Situated near the city of Hunchun, with a human population of approximately 250,000, this region serves as a critical ecological link between the Northeast China Tiger and Leopard National Park (NCTLNP) and the Changbai Mountains in China via North Korea, effectively bridging the habitats in the middle and upper reaches of the Tumen River (see Figure 1). Notably, this area provides a suitable habitat of 9149 km^2^ for the Far Eastern leopard [7]. The region experiences significant human activities, including the grazing of cattle, horses, and sheep; agriculture involving fruit orchards and ginseng cultivation; pig farming; tree frog farming; the operation of power plants; and the presence of concentrated cemeteries. 

However, the Hun-Wu Expressway bisects the forested landscape of the region and a high-speed railway. The Hun-Wu Expressway is particularly noteworthy, as both sides of the expressway feature fences rising to a height of 2.5 m, effectively preventing the direct crossing of large mammalian species. Within this transportation network, the “Mijiang Tunnel” is located within the Hun-Wu Expressway in the Mijiang area and serves as a roadway tunnel. Above this tunnel, the forested areas on both sides of the expressway are interconnected, creating a somewhat constricted natural wildlife corridor. Spanning approximately 500 m in width and 300 m in length, this corridor provides a safe passage for animals, shielding them from direct interaction with vehicular traffic.

**Figure 2:**
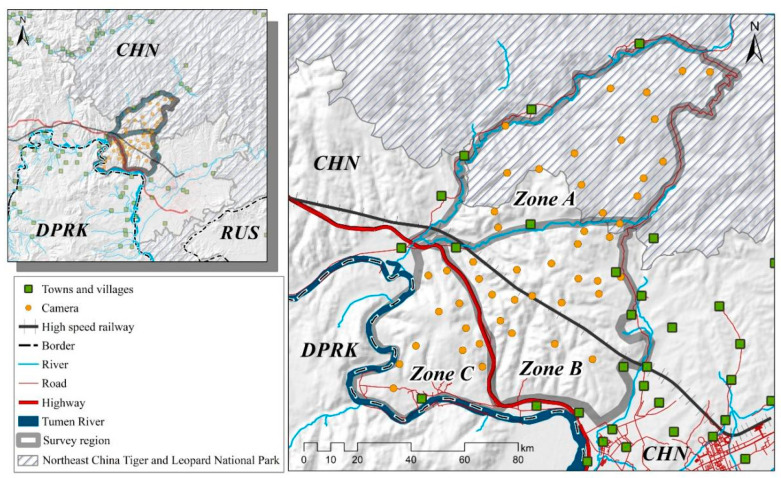
The study area’s map displaying camera trap locations, physical barriers, and human settlements. Note: All layers have been processed using ArcMap 10.8 (desktop.arcgis.com, accessed on 2 July 2023; ESRI, Redlands, USA).

The study area (MJ forest corridor) was divided into three distinct zones based on the straight-line distance from the Sino-Korea border, roads, and villages. Zone A represents the area adjacent to the NCTLNP, extending to the forests separated by the valleys and towns. Zone B is the central region adjacent to villages divided by the highway and valleys. Zone C corresponds to the forests adjacent to North Korea, extending from the Tumen River to the highway-separated forest. These three zones form a belt-shaped pattern, with the MJ forest corridor surrounded by neighboring settlements. The Amur leopard can only move from Zone A through Zone B to reach Zone C (see Figure 2).

### 2.2. Camera Trapping

Between March 2018 and July 2021, we installed 60 infrared cameras (HuntCam 3601, Zhuhai Ltl Acorn Electronics Co, Shenzhen, China) along the MJ tunnel highway. The camera locations were chosen to ensure a relatively uniform coverage of the study area. They were placed at approximately 1 km intervals at the intersections of frequently used wildlife tracks within the forest, including ridge lines, valleys, riversides, and forest paths in wetland areas. The average distance between cameras was 1404 m, ranging from 768 to 2774 m. All locations were carefully selected to avoid human settlements and roads [21].

We opted for the video format (mp4) for data capture, with a time interval of 1 min and a recording duration of 15 s upon triggering. Data management involved manual identification and annotation, with species and individual counts added to the video file names. The image names were used for the batch extraction of additional information, including date, time, species, and individual count [22]. Data were processed using an independent R code developed by Wildlife Coexistence Lab, UBC, in 2021 (https://bookdown.org/c_w_beirne/wildCo-Data-Analysis/, 5 April 2023). The time interval was adjusted to 30 min to categorize events as separate occurrences.

A relative abundance value (RAI) was calculated as RAI = (Ai/N) × 100, where Ai refers to the number of independent photos for a specific species, and N represents the total number of days the cameras were in operation (sum of the total number of days each camera was operational; summed for all cameras) [21,23]. RAIs were calculated for the Amur leopard, roe deer (*Capreolus pygargus*), sika deer (*Cervus nippon*), water deer (*Hydropotes inermis*), and wild boar (*Sus scrofa*), as well as for human activities and grazing in the three study zones. The RAI served as a measure of species abundance and disturbance intensity. The number of independent videos for each event was considered an indicator of species abundance. Here, ‘event’ refers to the specific types of occurrences captured by the cameras. 

### 2.3. Factor Process

#### 2.3.1. Vegetation Data Collection

Considering factors such as vegetation and biological community characteristics in the region, we chose 20 m × 20 m camera trapping grid points for environmental variable surveys. The cameras were ideally positioned in the central area of each grid point, though not strictly, to ensure the environmental representativeness of the surroundings near the camera [24]. The coordinates (latitude and longitude), elevation, slope, and aspect of the sampling plots were recorded using a GPS device (UniStrong, G138BD, Nanjing, China), along with a compass (SUUNTO, MC.2, Vantaa, Finland). Canopy closure and leaf area index (LAI) were determined using a canopy analysis instrument (Delta-T HemiView, Cambridge, UK). Within each sampling plot, three random measurement points were selected. Average values of the measurements were calculated to represent the canopy data for each plot. Photos of the canopy were taken using a fisheye lens camera placed at a height of 1.3 m. Photography sessions occurred during clear-sky conditions, specifically at sunrise (8:00–10:00 h) and sunset (15:00–17:00 h), to minimize direct sunlight and ensure sufficient light.

The canvas method was employed to assess understory transparency [25]. An orange waterproof canvas measuring 1.5 m × 1 m was placed 10 m from the plot’s center. The photographer stood at the center and captured photographs of the understory vegetation from four directions (east, south, west, and north) using an 80 mm focal length lens at a height of 1.3 m. All photos were converted to binary (black and white) raster images using software Sidelook version 1.1.01 [24]. These images were then imported into Gap Light Analyzer software (Version 2.0) (Simon Fraser University, Burnaby, British Columbia, Canada, USA) to calculate canopy closure, leaf area index, and understory transparency.

In the vicinity of the camera point, a 20 m × 20 m area was selected and scanned using lidar equipment (PARACOSM PX-80 HANDHELD MOBILE LiDAR, PX-80, Topsfield, MA, USA). Using the LIDAR360 software, we classified and extracted information on tree species, shrub coverage, tree height, and leaf area index [26]. Similarly, a manual vegetation survey was conducted within a 20 m × 20 m area near the camera point to record transparency, concealment, and the diversity and quantity of herbs, shrubs, and trees. The collected data were subsequently organized and analyzed using electronic spreadsheets [24].

#### 2.3.2. Terrain, Land Use, and Landscape Factors

Terrain data, encompassing elevation, slope, and aspect, were procured from the ASTER Global Digital Elevation Model (ASTER GDEM) at a 30 m resolution, available at https://www.jspacesystems.or.jp/ersdac/GDEM/E/, 10 August 2022. The study area was subdivided into 1 km × 1 km grids, and elevation, slope, and aspect data were extracted using the Extract tool within ArcGIS 10.8 software (ESRI, Redlands, CA, USA). Subsequently, the extracted data were categorized using the Reclassify tool. Altitude was classified into three groups: low altitude (0–200 m asl), mid-altitude (200–500 m asl), and high altitude (>500 m asl). The proportion of each category was calculated within each 1 km × 1 km grid. The slope was classified into three categories: gentle slope (0–10°), moderate slope (10–30°), and steep slopes (>30°), with the proportion of each category calculated within each grid. Aspect was categorized into three groups: shady slopes (315–360° and 0–45°), mixed shady and sunny slopes (45–135° and 225–315°), and sunny slopes (135–225°) [27,28].

Global land cover and land use data from 2019 (v1.0) were employed, featuring a 25 m resolution, accessible at https://glad.umd.edu/dataset/global-land-cover-land-use-v1, 10 August 2022. These data were reclassified into six categories following research requirements, utilizing the Reclassify tool in ArcGIS 10.8 software. The redefined categories included short vegetation, open mixed forest, dense mixed forest, wetland sparse vegetation, cropland, and infrastructure (urban residential land). The study area is primarily dominated by broadleaf forests, interspersed sporadically with mixed coniferous–broadleaf woodlands. The density of forest trees can influence animals in terms of visibility, concealment, and mobility [29]. In this context, data from sparse and dense forests were chosen as they reflect the leopard’s habitat preferences [30]. In this region, grazing in the forest is practiced seasonally. In the spring, livestock are driven to designated forest areas for free-range grazing and are herded back to individual small enclosures by autumn. Similar to the terrain data, the study area was divided into 1 km × 1 km grids, and the proportion of each land use category was calculated within each grid.

The study area was also segmented into 1 km × 1 km grids for landscape fragmentation analysis. Fragstats 4.2 software was employed to derive landscape metrics for each grid based on land use factors. Five landscape metrics were selected, including Patch Density (PD), Aggregation Index (AI), Landscape Shape Index (LSI), Contagion Index (CONTAG), and Shannon’s Diversity Index (SHDI) [31,32,33,34] (Table S1).

#### 2.3.3. Residential and Road Interference Intensity

In Northeast China, large mammals tend to avoid farmlands, railways, and roads [33]. We employed vector data representing residential areas and road networks to gauge the interference intensity of the residential areas and roads on camera monitoring points. The analysis was conducted using buffer analysis and interpolation tools within ArcGIS 10.8 software to create heatmaps illustrating the intensity of interference distances between camera points and residential areas or roads. Subsequently, numerical values were extracted for each camera point.

To evaluate residential area interference, we categorized the distances between residential areas and camera monitoring points and assigned interference intensity values. Similarly, for road interference, we considered road classifications and their distances from camera sites, assigning corresponding interference intensity values [35,36] (See Tables S2 and S3 for details).

#### 2.3.4. Statistical Analysis

The habitat selection and distribution of leopards are influenced by topographical factors, including slope, elevation, and river valleys [7,19,37]. Studies indicate that leopards tend to avoid human settlements, roads, deciduous forests, grasslands, shrublands, and farmlands [9,38,39]. Habitat fragmentation poses a threat to the stability of leopard habitats [5,40,41], and vegetation structure influences leopard habitat selection, including factors such as prey density, hunting convenience, and shelters [37,42,43,44]. Based on this, we considered a total of 33 factors, including topographical structure (9 factors), land use (6 factors), landscape fragmentation indices (5 factors), roads (1 factor), human settlements (1 factor), and vegetation structure (11 factors). To assess the variation among 33 selected factors within the Mijiang forest corridor’s three regions, we conducted an ANOVA test using Origin 2021 software. This analysis enabled us to calculate disparities in population variances, determine fit statistics, conduct tests for variance homogeneity (specifically Levene’s test with absolute deviation), and create box plots to compare the factors across the three zones [45].

## 3. Results

Out of the initial 60 camera traps, 48 were used for data analysis due to non-functional or lost cameras during the study period. A total of 48 operational cameras were distributed across the research zones, with 16 cameras in Zone A, 20 cameras in Zone B, and 12 cameras in Zone C. The total operation duration of the camera traps was 29,626 trap days, with the shortest deployment period being 228 days and the longest being 792 days. In total, 19,656 valid shots were recorded, capturing 18 species of mammals including Amur tiger (*Panthera tigris altaica*), Amur leopard (*Panthera pardus orientalis*), Leopard cat (*Prionailurus bengalensis euptilurua*), Asian black bear (*Ursus thibetanus*), Red fox (*Vulpes vulpes*), Raccoon dog (*Nyctereutes procyonoides*), Asian badger (*Meles leucurus*), Siberian weasel (*Mustela sibirica*), Yellow-throated marten (*Martes flavigula*), Eurasian otter (*Lutra lutra*), Wild boar (*Sus scrofa*), Roe deer (*Capreolus pygargus*), Sika deer (*Cervus nippon*), Water deer (*Hydropotes inermis*), Siberian chipmunk (*Tamias sibiricus*), Eurasian red squirrel (*Sciurus vulgaris*), Amur hedgehog (*Erinaceus amurensis*), and Manchurian hare (*Lepus mandshuricus)* (Table S4). 

**Figure 3:**
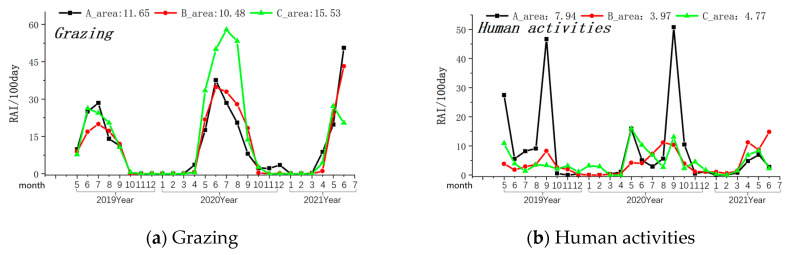
Monthly variations in the intensity of the grazing and human activity. Note: The axis at the bottom of the figure represents the months, while the left axis represents the RAI value over 100 days. The values displayed at the top of the figure represent the RAI values over a total of 100 days during the monitoring period in areas A, B, and C. The black line indicates changes in area A, the red line indicates changes in area B, and the green line indicates changes in area C.

The Amur leopard exhibited its highest presence in Zone A (Relative Abundance Index/100 days-0.17), was rarely seen in Zone B (Relative Abundance Index/100 days-0.03), and was not observed in Zone C of the MJ forest corridor. Grazing activity was most frequently observed in Zone C, followed by Zone A and Zone B (see Table 1). Other human activities (forest produce collection, farming, logging, and visitors with cars) were most prevalent in Region A (Relative Abundance Index/100 days-7.94) and least in Region B (Relative Abundance Index/100 days-3.97). Grazing peaks occurred between May and June in all three Zones (see Figure 3). Similarly, the intensity of other human activities was highest during May and September in all the studied Zones (see Figure 3).

**Figure 4:**
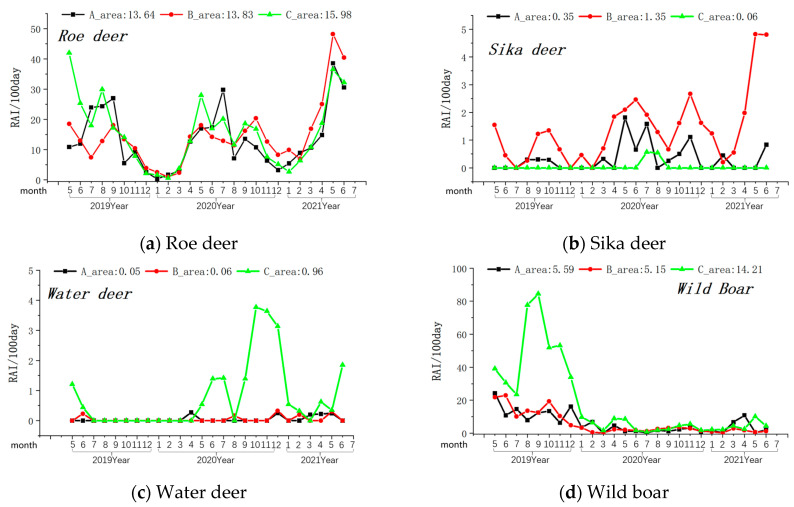
Monthly variations in the relative abundance of four primary prey species of the Amur leopard. Note: The axis at the bottom of Figure represents the months, while the left axis represents the RAI value over 100 days. The values displayed at the top of the figure represent the RAI values over a total of 100 days during the monitoring period in areas A, B, and C. The black line indicates changes in area A, the red line indicates changes in area B, and the green line indicates changes in area C.

Among the prey species, the overall relative abundance (Relative Abundance Index/100 days) was highest for roe deer, followed by wild boar, sika deer, and water deer (see Table 1). In the monthly variation curves of the Relative Abundance Index values for prey resources, the relative abundance of wild boar and water deer was consistently higher in Zone A, while sika deer’s abundance was highest in Zone B (see Figure 4).

**Figure 5:**
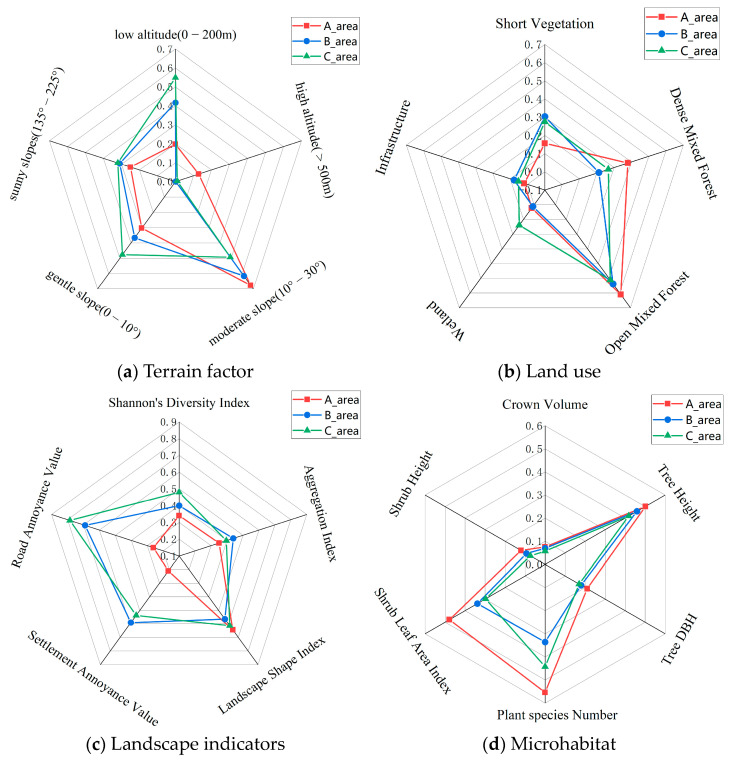
Schematic diagram of the classification of 22 factors with significant differences across the three Zones (A, B, and C) of the MJ tunnel corridor.

Out of the 33 factors (terrain, land use, landscape, and microhabitat) assessed, 22 factors showed significant differences (*p* ≤ 0.01) across the three Zones (see Table S5). The data for these 21 factors with significant differences were normalized, resulting in values between 0 and 1. A grid diagram representing these normalized values is shown in Figure 5. 

The Amur leopard exhibits a preference for broad-leaved and mixed coniferous–broadleaf forests, with an activity range of 200–1800 m in elevation [9,37]. We speculate from our captures that leopards tend to use ridges and open forest steep cliffs [46]. Significant differences exist in the habitat structure across the tunnel corridor zones (see Figure 5). More than 80% of the habitat in Zone A was above 200 m asl compared to 58% and 45% in Zone B and C. Sunny slopes (135–225°) had the highest percentage in Zone C (28.6%), followed by Zone B (27.7%) and Zone A (22.3%). Zone A had the highest proportion of open mixed forest and dense mixed forest and the least amount of cropland. Mean tree height, diameter at the breast (DBH), and crown volume were highest in Zone A (11.3 m, 13.9 cm, 64.7 m^3^), followed by Zone B (10.6 m, 12.4 cm, 59.7 m^3^), and Zone C (9.8 m, 11.8 cm, 49.1 m^3^). Similarly, the mean shrub tree height and leaf area index were highest in Zone A (99.1 cm, 2.63), followed by Zone B (77.6 cm, 1.05), and Zone C (61.6 cm, 0.77). Landscape fragmentation was lowest in Zone A (Shannon diversity index-0.86; Aggregation Index: 78.41; Settlement Annoyance Value: 4.97; and Road Annoyance Value: 1.18). Species richness was highest in Zone A, followed by Zone C and Zone B.

## 4. Discussion

The Amur leopard, classified as critically endangered, has seen its habitat and distribution range significantly reduced. Historically, it was widespread in the Korean peninsula but has suffered regional extinction primarily due to unchecked hunting and habitat degradation. The prospects for the species’ recovery in the wild are closely tied to its restoration in the Korean peninsula, which constitutes a substantial portion of its historical habitat [1].

The Tumen River basin, situated in the Sino-Korean border region, plays a crucial role in the potential recovery of the Amur leopard population in the Korean peninsula. The downstream areas of the Tumen River boast the highest Amur leopard density in China, with recent studies highlighting cross-border animal movements in this region. However, human settlements, farming, grazing, and extensive transportation networks such as highways and railways have led to the significant fragmentation of local forests. In this context, the MJ tunnel corridor represents one of the last remaining forest connections between Northeast China and the Korean peninsula.

Our study offers comprehensive insights into the distribution and densities of Amur leopards and their potential prey species within the Mijiang region. Additionally, we have examined landscape types, vegetation types, and human disturbances. This information is vital for devising long-term management strategies aimed at restoring leopard populations in the Sino-Korean border regions and, potentially, the Korean peninsula.

The availability of prey resources stands out as a critical factor influencing the distribution and reproductive success of large carnivores [12,47]. In the forested areas of the MJ corridor, we identified several prey species, including roe deer, sika deer, wild boar, water deer, badgers, raccoon dogs, red foxes, Manchurian hares, and cattle. In the Sino-Russia border regions, roe deer, sika deer, and wild boar are the primary prey species for Amur leopards [47]. The relative abundance (RAI) of these prey species, except for sika deer, was higher in the Mijiang region compared to the current distribution boundaries of leopard populations in the Sino-Russia border regions [47,48]. This suggests that there is a sufficient prey base to support leopard survival and reproduction in the Mijiang region.

The leopard activity was predominantly confined to Zone A of the corridor. In the Mijiang region, Zone A offers better survival conditions for the Amur leopard, characterized by a higher proportion of complex forests, and optimal elevations (200–1800 m) compared to the other two Zones. Additionally, landscape fragmentation and cropland areas were found to be lowest in Zone A. However, in Zone A, the scarcity of sunny slopes might be the factor limiting leopard appearances during winter in the area, potentially influenced by the deeper snow caused by the slope [9].

However, the Mijiang region as a whole experiences very high intensities of human activity and grazing. These factors may hinder Amur leopards from moving from Zone A to Zone C through Zone B. Human disturbances and grazing activities are known to have restricted the Amur leopard to a narrow region along the China-Russia border [5]. Therefore, the high levels of human activity and grazing in the Mijiang region could potentially impact the movement and distribution of these critically endangered animals, underscoring the need for careful management and conservation efforts.

Numerous studies have highlighted the potential detrimental effects of domestic dogs’ presence in forested environments on wildlife as dog behavior (barking and chasing to hunt) disturbs feeding, breeding, and the survival of wildlife [49,50]. Another concern that merits attention is the role of domestic dogs as potential vectors for diseases such as rabies and canine distemper. These diseases pose latent risks and can be transmitted from dogs to wildlife, thus intensifying the threats to wildlife ecology [51]. Within our study area, we recorded 112 instances of dog activity, with Area A registering the highest number of occurrences at 51. However, it is significant to note that these instances of dog activity were relatively few compared to human activities. Additionally, dietary studies within the Tiger-Leopard Park did not find evidence of leopards preying on dogs [47]. This may be because there are few dogs in this region available to be preyed upon (Table 1). Closer observation revealed that most captured instances of dog activity were accompanied by human presence, with solitary appearances of dogs being rare. Based on these observations, we tentatively infer that, within this region, the ecological impact of dogs on the Amur leopard is relatively minimal. Nevertheless, the potential role of dogs in disease transmission to Amur leopards cannot be discounted. This area warrants further research, particularly more in-depth studies on local dogs and leopards, to ascertain their precise impact on the Amur leopard.

## 5. Conclusions

Human activities and urbanization have accelerated habitat fragmentation and isolation in the Tumen River basin [52]. Residential areas and roads have had adverse effects on leopard behavior, hindering their movement, migration, and breeding [53]. Along the China–Russia border, areas farther from residential areas and roads have significantly higher leopard numbers [12]. The Mijiang area is currently surrounded by numerous villages, with several smaller settlements within its boundaries. It remains an unprotected area with extensive human activities, including grazing, farming, logging (often on a small scale for winter heating), ginseng cultivation, burial grounds, poaching, and the presence of domestic dogs [54]. The protective fences on either side of the highway and high-speed railway have disrupted animal movement across zones, with the exception of a 400-m-wide forest section atop the Mijiang tunnel. Unrestricted leopard movement in the Tumen River basin is vital for the long-term survival of the Amur leopard, enabling them to migrate to potential alternative habitats like the Changbai mountain region in China and North Korea. Consequently, we recommend a reduction in human activities by engaging local communities in alternative livelihoods and curtailing further infrastructure-driven habitat destruction. We also propose the construction of additional eco-bridges and underpasses across highways and rail networks to facilitate free animal movement.

Large felids like tigers and leopards require extensive habitats with mature forests for their survival, expansion, and reproduction [2]. Complex structures within such habitats offer shelter for hiding, resting, breeding, access to water sources, prey, and potential mates [55]. Deforestation and logging disrupt forest structure, animal movement, and reproductive success [56]. Zone A of the tunnel corridor has a relatively higher percentage of mature forests compared to the other two Zones, and we observed most leopard activity in Zone A. Thus, activities aimed at forest restoration and restrictions on logging are recommended to ensure forest regeneration.

The population restoration of large carnivores such as leopards and tigers contributes to the revival of forests and other wildlife species. Healthy forests ensure a clean environment, maintain water resources, and prevent soil erosion. Collaborative efforts are crucial to secure the promising future of the Amur leopard. Through this research, we aim to garner increased attention from various segments of society, including local communities, government agencies, non-governmental organizations, and international entities. It is vital to prevent further habitat degradation in the Tumen River basin by adopting eco-sensitive development and occupational approaches. Once protected and restored, the river basin can facilitate wildlife movement across borders. Safeguarding the ecological corridor in the downstream Tumen River is indispensable to ensure that large felids like the Amur leopard can reestablish their populations on the Korean Peninsula in the coming decades.

## Figures and Tables

**Table 1:** Statistical summary of species abundance and disturbances in the Mijiang region (Camera-trapping). Note: frequency: no. of independent photos; RAI per 100 Trap-Nights: the sum of the number of independent images from all camera sites divided by the total actual operating days of all cameras in the area, multiplied by 100 days.

Detection (Photo-Capture)	Frequency/RAI per 100 Trap-Nights		
	Zone A	Zone B	Zone C
Amur leopard	17/0.17	4/0.03	0/0
Sika deer	35/0.35	168/1.35	4/0.06
Roe deer	1376/13.64	1718/13.83	1137/15.98
Wild boar	564/5.59	640/5.15	1011/14.21
water deer	5/0.05	6/0.05	68/0.96
Other human activities	801/7.94	493/3.97	339/4.77
Grazing	1175/11.65	1302/10.48	1105/15.53
Dog	51/0.51	36/0.29	25/0.35

## Data Availability

The datasets generated and/or analyzed during this current study are available from the corresponding author upon reasonable request. The data are not publicly available due to joint monitoring project contract.

## Supplementary Materials

The following are available online at https://www.mdpi.com/article/10.3390/ani14010059/s1, Table S1: List of selected landscape metrics for landscape fragmentation analysis; Table S2: Interference intensity value based on the distance between residential sites and camera points; Table S3: Interference intensity value based on the distance between roads and camera points; Table S4: The number of valid incidents involving animals, human activities, grazing, and dogs captured by camera traps in the Mijiang area; Table S5: One way ANOVA analysis of 33 environmental factors across three regions of the Mijiang area.
