# Supplementary material for: Transboundary Cooperation in the Tumen River Basin Is the Key to Amur Leopard (Panthera pardus) Population Recovery in the Korean Peninsula
Source: Animals (Basel). 2023 Dec 22;14(1):59. doi: 10.3390/ani14010059 (PMC10778315; doi:10.3390/ani14010059)
Supplement: Supplementary file 1 [file animals-14-00059-s001.zip › animals-2694123-supplementary.pdf]

SUPPLEMENTARY MATERIALS

Table S1. List of Selected Landscape Metrics for Landscape Fragmentation Analysis

Landscape index	Abbreviation	Description
Patch Density	PD	Represents the number of patches per unit area. Higher values indicate higher landscape fragmentation
Aggregation Index	AI	Represents the percentage of shared edges between adjacent patches of the same class. Higher values indicate higher aggregation of dominant patches.
Landscape Shape Index	LSI	Represents the ratio of total edge length to total patch area. Higher values indicate more complex and fragmented patch shapes.
Contagion Index	CONTAG	Assess the connectivity or continuity of different land cover classes in the landscape. A higher Contagion Index value indicates that different land cover types are more contiguous and less fragmented, meaning that they are closely connected or adjacent to each other.
Shannon's Diversity Index	SHDI	Used to quantify the diversity of species within a particular habitat or ecosystem. Index takes into account both the number of different species (species richness) and their relative abundance. A higher Shannon's Diversity Index value indicates greater diversity, where more species are present, and the community is relatively evenly distributed in terms of species abundance. Conversely, a lower index value suggests lower diversity, either due to fewer species or an uneven distribution of species.

Table S2. Interference intensity value based on the distance between residential sites and camera points.

Distance from resident sites	0-1km	1-3km	3-5km	>5km
Weight value	1	0.8	0.3	0

Table S3. Interference intensity value based on the distance between roads and camera points

Road type	1km	3km	5km	10km
High way	1	0.8	0.3	0.1
Highspeed railway	1	0.8	0.3	0.1
Provincial road	0.8	0.3	0.1	0
Secondary road	0.3	0.1	0	0
Railway	0.3	0.1	0	0

Table S4. The number of valid incidents involving animals, human activities, grazing, and dogs captured by camera traps in the Mijiang area.

N0.	Family	Species	Scientific Name	IUCN	CITES	Count	frequency/RAI per 100 Trap-Nights		
							Zone A	Zone B	Zone C
1	Felidae	Amur tiger	*Panthera tigris altaica*	EN	I	1	0.01	0	0
2	Felidae	Amur leopard	*Panthera pardus orientalis*	CR	I	21	0.17	0.03	0
3	Felidae	Leopard cat	*Prionailurus bengalensis euptilurua*	LC	II	93	0.21	0.25	0.58
4	Ursidae	Asian black bear	*Ursus thibetanus*	VU	I	7	0.04	0.02	0.01
5	Canidae	Red fox	*Vulpes vulpes*	LC		898	2.55	3.66	2.61
6	Canidae	Raccoon dog	*Nyctereutes procyonoides*	LC		1248	3.1	5.89	16.26
7	Mustelidae	Asian badger	*Meles leucurus*	LC		2202	5.65	3.37	3.64
8	Mustelidae	Siberian weasel	*Mustela sibirica*	LC		119	0.34	0.61	0.13
9	Mustelidae	Yellow throated marten	*Martes flavigula*	LC		104	0.51	0.35	0.13
10	Mustelidae	Eurasian otter	*Lutra lutra*	NT	I	1	0	0.01	0
11	Suidae	Wild boar	*Sus scrofa*	LC		2475	5.59	5.15	14.21
12	Cervidae	Roe deer	*Capreolus pygargus*	LC		4419	13.64	13.83	15.98
13	Cervidae	Sika deer	*Cervus nippon*	LC		210	0.35	1.35	0.06
14	Cervidae	Water deer	*Hydropotes inermis*	VU		81	0.05	0.05	0.96
15	Sciuridae	Eurasian red squirrel	*Sciurus vulgaris*	LC		25	0.09	0.09	0.07
16	Sciuridae	Siberian chipmunk	*Tamias sibiricus*	LC		11	0.08	0.02	0
17	Erinaceidae	Amur hedgehog	*Erinaceus amurensis*	LC		65	0.32	0.19	0.13
18	Leporidae	Manchurian hare	*Lepus mandshuricus*	LC		338	0.84	0.67	2.39
19		Other human activities				1633	7.94	3.97	4.77
20		Grazing				3582	11.65	10.48	15.53
21		Dog				112	0.51	0.29	0.35

Table S5. One way ANOVA analysis of 33 environmental factors across three regions of the Mijiang area

Category	Content	Zone A		Zone B		Zone C		F value	P value	R^2_value
		Mean	SE	Mean	SE	Mean	SE			
Landscape indicator	Settlement disturbance	4.97^b	0.36	9.16^a	0.36	8.53^a	0.24	43.63	0.000	0.66
Microhabitat	Shrub Height（cm）	99.05^a	3.05	77.59^b	2.36	61.62^c	2.28	42.98	0.000	0.03
Microhabitat	Tree Height (m）	11.33^a	0.15	10.55^b	0.11	9.79^c	0.14	30.61	0.000	0.03
Land use	Wetland (%)	0.61^b	0.21	0.32^b	0.13	3.78^a	0.82	22.57	0.000	0.13
Terrain factor	Elevation（0~200m）	19.82^b	2.86	41.72^a	3.92	54.92^a	5.23	21.26	0.000	0.12
Landscape indicator	Road disturbance	1.18^b	0.17	2.28^a	0.13	2.52^a	0.16	21.04	0.000	0.48
Terrain factor	Elevation（>500m）	12.87^a	2.28	0.16^b	0.13	0.98^b	0.48	19.27	0.000	0.11
Microhabitat	Tree DBH (cm)	13.92^a	0.30	12.39^b	0.23	11.79^b	0.27	16.34	0.000	0.01
Land use	Short Vegetation	12.61^b	1.20	24.42^a	1.84	22.08^a	2.31	16.03	0.000	0.10
Terrain factor	Slope（10~30°）	61.82^a	1.55	56.39^a	2.33	45.34^b	2.44	13.74	0.000	0.08
Landscape indicators	SHDI	0.86^b	0.02	0.93^b	0.02	1.04^a	0.03	13.56	0.000	0.08
Land use	Dense forest	29.51^a	1.87	16.50^b	1.76	20.76^b	2.54	12.84	0.000	0.08
Terrain factor	Slope（0~10°）	33.67^b	1.64	39.60^b	2.52	49.91^a	2.69	11.54	0.000	0.07
Landscape indicators	AI	78.41^b	0.29	80.86^a	0.47	79.68^{ab}	0.47	10.86	0.000	0.07
Landscape indicators	LSI	5.45^a	0.06	5.02^b	0.09	5.29^{ab}	0.09	9.21	0.000	0.06
Microhabitat	Crown Volume	64.66^a	3.30	59.73^a	2.34	49.13^b	2.15	7.99	0.000	0.01
Terrain factor	Elevation（200~500m）	67.31^a	3.07	58.12^{ab}	3.91	44.10^b	5.09	7.96	0.000	0.05
Land use	Infrastructure	1.59^b	0.49	6.57^a	1.39	4.49^{ab}	1.04	7.29	0.001	0.05
Microhabitat	Shrub leaf area index	2.63^a	0.61	1.05^b	0.19	0.77^b	0.16	7.08	0.002	0.25

Table S5. One way ANOVA analysis of 33 environmental factors across three regions of the Mijiang area (continued)

Category	Content	Zone A		Zone B		Zone C		F value	P value	R^2 value
		Mean	SE	Mean	SE	Mean	SE			
Microhabitat	Plant species number	13.19^{a}	1.18	8.40^{b}	0.78	10.75^{ab}	1.47	5.41	0.008	0.19
Land use	Open forest	53.66^{a}	1.43	47.32^{b}	2.21	44.98^{b}	2.60	5.19	0.006	0.03
Terrain factor	Sunny slope (135°-225°)	22.30^{b}	1.25	27.66^{a}	1.23	28.57^{a}	2.58	5.17	0.006	0.03
Land use	Cropland	2.02^{b}	0.55	4.86^{a}	1.07	3.90^{ab}	1.12	3.17	0.043	0.02
Microhabitat	Number of trees	141.8	20.3	88.65	12.3	126.3	20.28	2.86	0.067	0.11
Landscape indicators	PD (Patch Density)	45.97	0.81	42.89	1.13	43.89	1.19	2.77	0.065	0.02
Landscape indicators	CONTAG (Contagion Index)	42.62	0.67	45.23	0.92	43.66	1.22	2.58	0.078	0.02
Microhabitat	Permeability	17.25	0.44	18.45	0.48	17.14	0.51	2.42	0.093	0.03
Terrain factor	Mixed shady and sunny slopes (45°-135° and 225°-315°)	51.84	1.27	48.27	1.60	48.03	1.98	2.07	0.129	0.01
Terrain factor	Tree leaf area index	3.70	0.21	4.20	0.26	4.36	0.29	1.53	0.228	0.07
Terrain factor	shady slopes (315°-360° and 0°-45°)	25.83	1.45	24.02	1.75	21.29	2.26	1.47	0.233	0.01
Microhabitat	Concealment	66.53	3.58	71.40	2.99	72.19	3.54	0.82	0.440	0.01
Terrain factor	Slope（>30°）	4.51	0.47	4.01	0.46	4.75	0.86	0.42	0.658	0.00
Microhabitat	Number of shrubs	220.6	43.8	257.5	98.2	312.3	112.0	0.24	0.788	0.01

*The data is arranged in ascending order based on the p-value (a, b, c are the groupings from Tukey's test).

(a)Topographic factors: Low altitude (0~200m) – 19.8% (A), 41.7% (B), 54.9% (C); High altitude (>500m) – 12.9% (A), 0.2% (B), 1% (C); Gentle slope (0~10°) – 33.7% (A), 39.6% (B), 49.9% (C); Moderate slope (10~30°) – 61.8% (A), 56.4% (B), 45.3% (C); Sunny slopes (135°-225°) – 22.3% (A), 27.7% (B), 28.6% (C)(Table S5).

(b)Land use: Shrub Vegetation – 12.6% (A), 24.4% (B), 22.1% (C); Open Mixed Forests – 53.7% (A), 47.3% (B), 45.0% (C); Dense Mixed Forests – 29.5% (A), 16.5% (B), 20.8% (C); Infrastructure – 1.6% (A), 6.6% (B), 4.5% (C) (Table S5).

(c)Landscape indicators: Shannon's Diversity Index – 0.86 (A), 0.93 (B), 1.04 (C); Aggregation Index – 78.41 (A), 80.86 (B), 79.68 (C); Landscape Shape – 5.45 (A), 5.02 (B), 5.29 (C); Settlement Annoyance Value – 4.97 (A), 9.16 (B), 8.53 (C); Road Annoyance Value – 1.18 (A), 2.28 (B), 2.52 (C)(Table S5).

(d)Microhabitat: Tree Height – 11.3m (A), 10.6m (B), 9.8m (C); Tree DBH – 13.9cm (A), 12.4cm (B), 11.8cm (C); Crown Volume – 64.7m^3 (A), 59.7m^3 (B), 49.1m^3 (C); Shrub Height – 99.1cm (A), 77.6cm (B), 61.6cm (C); Shrub Leaf Area Index – 2.63 (A), 1.05 (B) , 0.77 (C); Plant species Number – 13.2 (A), 8.4 (B), 10.8 (C)(Table S5).
